# A Hybrid Structure Learning Algorithm for Bayesian Network Using Experts’ Knowledge

**DOI:** 10.3390/e20080620

**Published:** 2018-08-20

**Authors:** Hongru Li, Huiping Guo

**Affiliations:** Information Science and Engineering, Northeastern University, P.O. Box 135, No. 11 St. 3, Wenhua Road, Heping District, Shenyang 110819, China

**Keywords:** Bayesian network, structure learning, explicit knowledge, vague knowledge, hybrid algorithm

## Abstract

Bayesian network structure learning from data has been proved to be a NP-hard (Non-deterministic Polynomial-hard) problem. An effective method of improving the accuracy of Bayesian network structure is using experts’ knowledge instead of only using data. Some experts’ knowledge (named here explicit knowledge) can make the causal relationship between nodes in Bayesian Networks (BN) structure clear, while the others (named here vague knowledge) cannot. In the previous algorithms for BN structure learning, only the explicit knowledge was used, but the vague knowledge, which was ignored, is also valuable and often exists in the real world. Therefore we propose a new method of using more comprehensive experts’ knowledge based on hybrid structure learning algorithm, a kind of two-stage algorithm. Two types of experts’ knowledge are defined and incorporated into the hybrid algorithm. We formulate rules to generate better initial network structure and improve the scoring function. Furthermore, we take expert level difference and opinion conflict into account. Experimental results show that our proposed method can improve the structure learning performance.

## 1. Introduction

Bayesian networks (BN) is one of the most effective theoretical models for decision making, especially for uncertain knowledge reasoning [1]. In recent years, Bayesian networks have been widely used in a variety of domains such as medical diagnosis [2], device fault diagnosis [3], and system modeling in multiple situations [4,5,6]. Bayesian network learning is the fundamental topic in Bayesian network research. Generally, Bayesian network learning consists of two parts, structure learning and parameter learning. Of these two parts, structure learning is the core part for Bayesian network learning.

Bayesian network structure learning is to determine every edge in the BN, including judging the existence of edges and the direction of edges. Generally, there are three main algorithms in Bayesian network structure learning: independence-based structure learning algorithms (independence-based algorithms) [7,8], score-based structure learning algorithms (score-based algorithms) [9,10], and hybrid structure learning algorithms (hybrid algorithms) [11,12,13]. The first group of algorithms use conditional independence (CI) tests to identify conditional independent relationships among variables. The major weakness of these algorithms is high complexity and sensitivity to the CI tests [14]. The second group of algorithms consider structure learning as a structural optimization problem, using a search strategy to select the structure with the highest score of a scoring function which measures the fitting degree of network and data. The major weakness of these algorithms is that they easily to fall into local optima. Both groups of algorithms have drawbacks. Recently, the third group of algorithms, hybrid algorithms, has become widely used because they combine the advantages of the previous two algorithms. One popular strategy is to use independence-based algorithms (the first stage learning algorithms) to determine the initial network structure and use score-based algorithms (the second stage learning algorithms) to find the highest-scoring network structure.

Bayesian network structure learning from data has been proved to be a NP-hard problem [15], and it faces great challenges when using structural learning algorithms. Firstly, the generation of the initial network structure is very sensitive to CI tests. If the data is insufficient or noisy, the algorithms may work incorrectly. Secondly, the number of the candidate structures grows exponentially with the increase in the number of variables. Finding the highest score network structure through the search strategy is very hard and the search strategy is easy to fall into local optimum. Thirdly, the scoring functions can’t work accurately with noisy or insufficient data. Finally, many network structures may have Markov equivalent graph structures that have same joint probability distributions and can’t be distinguished by data alone. The effective way to eliminate those limitations is to incorporate experts’ knowledge into Bayesian network structure learning.

During the last two decades, a lot of studies have been devoted to incorporating experts’ knowledge into Bayesian network structure learning [16,17,18,19]. The weakness in the majority of the proposed algorithms is that they assume that the knowledge given by experts can make the causal relationship between nodes in BN structure clear (explicit knowledge), but knowledge provided by experts that cannot make the causal relationship between nodes in BN structure clear (vague knowledge) also exists in the real world. For example, the experts may deem that there is a link between node A and node B. This expert knowledge can’t determine the orientation of this pair of nodes in BN. In other words, this expert knowledge is not explicit. We consider it necessary to take this kind of knowledge into consideration in the process of Bayesian network structure learning, rather than just using explicit knowledge alone. Therefore, how to incorporate these two types of experts’ knowledge into Bayesian network structure learning is a core problem. We propose a new hybrid algorithm with two types of experts’ knowledge to solve the core problem of the use of more extensive experts’ knowledge. Using multiple experts, we may also encounter problems of expert level difference and opinion conflict on the same group of node pairs. We incorporate the accuracy of expert into the proposed algorithm and use the principle of the obedience of minority to majority based on credibility to solve those two problems.

In this paper, we propose a new method of using more comprehensive experts’ knowledge based on hybrid structure learning algorithm for Bayesian network. We define three kinds of vague knowledge {↚,↛,−}, and three kinds of explicit knowledge  {→,←,↮} in this new method. Moreover, we take the accuracy of each expert into account by using six parameters, which we extend the accuracy of an expert in the field of explicit knowledge [17] to the field of both explicit and vague knowledge. The two types of experts’ knowledge and the accuracy of each expert are incorporated with training data in hybrid algorithm. In order to improve the utilization of knowledge, this paper applies knowledge to a two-stage hybrid algorithm. In the first stage, firstly, the credibility of experts’ knowledge is determined by six accuracy parameters. Then, we fuse the different experts’ knowledge of the same node pair based on credibility. Finally, we formulate rules to add and delete edges based on different types of experts’ knowledge in the process of generating the initial network structure. In the second stage, we propose an explicit-vague-Bayesian Information Criterion (EVBIC) scoring function which built upon the explicit-accuracy-based score function proposed by [17]. The main advantage of our score function is that it can use vague knowledge. Experimental results reveal that the proposed method can make good use of vague knowledge and explicit knowledge given by experts and can learn a better BN structure than hybrid algorithms.

The remainder of this paper is structured as follows: Section 2 introduces the notations and preliminaries that are used in subsequent sections. Section 3 describes the proposed method. Section 4 details our experimental procedures and presents the experimental evaluation of the algorithm. Finally, Section 5 concludes the work.

## 2. Preliminaries

In this section, a brief summary of the Bayesian networks and the hybrid structure learning algorithm is introduced. What’s more, we present two types of experts’ knowledge and they are subdivided into six kinds of experts’ knowledge, along with some further notations that will be used throughout the remainder of this paper.

### 2.1. Bayesian Network

A Bayesian network is a probabilistic graphical model based on probability theory and graph theory. A Bayesian network is a directed acyclic graph that describes the joint probability distribution over a set of random variables, with defining a series of probability independences and conditional independences [20]. A Bayesian network can be represented as a tuple BN=(G,ρ) where G=(V,E). G represents a structure known as a directed acyclic graph (DAG), with a set of nodes $V=\left\{X_{1},X_{2},\ldots X_{n}\right\}$ and a set of directed edges $E=\left\{<X_{1},X_{2}>,<X_{1,}X_{3}>,\ldots <X_{i},X_{j}>\right\}$. $X_{i}$ is a random variable. $<X_{i},X_{j}>\in E$ represents the directed connect between $X_{i},X_{j}$, noted as $X_{i}\to X_{j}$, with node $X_{i}$ known as the parent of node $X_{j}$, and node $X_{j}$ known as the child of node $X_{i}$. $\rho =\left\{\rho_{1},\rho_{2},\ldots ,\rho_{n}\right\}$ represents a set of conditional probability distributions of nodes. $\rho_{i}\in \rho$ represents the conditional probability distribution of node $X_{i}$. According the chain rule of Bayesian network, the Bayesian network represents the joint probability of all nodes that can be written as the product of the conditional probability distribution of each node:

(1) $$P\left(X_{1},X_{2},\ldots ,X_{n}\right)=\overset{n}{\underset{i=1}{\prod }}P(X_{i}|X_{1},X_{2},\ldots ,X_{i-1})$$

where n represents the number of nodes in Bayesian network, i=1,2,…,n.

Assuming that the conditional probability distribution of the node $X_{i}$ is only affected by its parent set $\pi \left(X_{i}\right)\subseteq \left\{X_{1},X_{2},\ldots ,X_{i-1}\right\}$, the Equation (1) can be written as:

(2) $$P\left(X_{1},X_{2},\ldots ,X_{n}\right)=\overset{n}{\underset{i=1}{\prod }}P(X_{i}|\;\pi \left(X_{i}\right))$$

where n represents the number of nodes in Bayesian network, i=1,2,…,n.

### 2.2. Hybrid Structure Learning Algorithms

As mentioned above, one category of Bayesian network structure learning algorithms is the hybrid structure learning algorithms. They aggregate both independence-based and score-based structure learning algorithms to give full play to the advantages of them and has been widely used at present. One popular strategy is using independence-based algorithms (the first stage learning algorithms) to determine the initial network structure and use it as a seed of score-based algorithms (the second stage learning algorithms) which include search strategies and scoring functions to find the highest score network structure.

#### 2.2.1. The First Stage Structure Learning Algorithms

The first stage structure learning algorithms generally use independence-based structure learning algorithms. The independence-based algorithms determine all the independence and dependence relationships among variables via conditional independence (CI) tests and construct networks that characterize these relationships. The independence-based algorithms can be divided into two processes. The first process is to determine whether the edges exist so that we can obtain the undirected network structure. The second process is to direct the edge orientations, especially to direct edges to form head-to-head patterns (triplets of nodes x,y,z such that x and y aren’t adjacent and the arcs x⟶z and y⟶z exist). In this paper, we use the Maximum Information Coefficient (MIC) and conditional independence tests to generate an initial network structure [21]. The initial network structure may still contain some undirected edges which will be used as an initial solution to the second stage structure learning algorithms.

#### 2.2.2. The Second Stage Structure Learning Algorithms

The second stage structure learning algorithms generally use score-based structure learning algorithms. The score-based algorithms have two main components: a search strategy and a scoring function. The number of possible DAGs is super-exponential in the number of random variables, given by the following function [22]:

(3) $$f\left(n\right)=f\left(x\right)=\{\begin{array}{cc} 1, & n=1\\ \overset{n}{\underset{i=1}{\sum }}{\left(-1\right)}^{i+1}\frac{n!}{\left(n-i\right)!\cdot i!}2^{i\left(n-i\right)}f\left(n-i\right), & n>1 \end{array}$$

It is obvious that the search space is huge. Therefore, heuristic search methods have been used to build the network structure, such as Genetic Algorithms, Ant Colony Optimization, Binary version of Particle Swarm Optimization (BPSO) [23]. In this paper, we use the BPSO as our search method. The BPSO utilizes a cooperative swarm of particles, where each particle represents a candidate solution to the problem (the possible DAGs), to explore the space of possible solutions (the searching space) to optimization of interest.

Scoring functions are usually used to measure the quality of the constructed BNs. There are variety of scoring functions such as the Bayesian Dirichlet equivalent uniform (BDeu) scoring function, the Minimum Description Length (MDL) scoring function and the Bayesian Information Criterion (BIC) scoring function, to mention a few. The detail of BIC scoring function is introduced blew since it is used in this study. BIC scoring function is in the form of a penalized log-likelihood (LL) function. Given a training dataset D, the log-likelihood function for a structure can written as:

(4) $$LL\left(D\right)=\overset{n}{\underset{i=1}{\sum }}\overset{r_{i}}{\underset{k=1}{\sum }}\overset{q_{i}}{\underset{j=1}{\sum }}m_{ijk}ln\frac{m_{ijk}}{m_{ij}}$$

where $m_{ijk}$ is the number of samples in the dataset that $X_{i}=k$ and its parents are in their *j*-th configuration, and likewise, $m_{ij}$ is the number of samples in the dataset that variable is *i*-th and its parents are in their *j*-th configuration. n is the number of random variables in Bayesian network, $r_{i}$ is the number of different states of *i*-th random variable, and $q_{i}$ is the number of possible configurations for parents of *i*-th random variable. A penalization is used and BIC scoring function can be written as follows:

(5) $$BIC\left(D\right)=\overset{n}{\underset{i=1}{\sum }}\overset{r_{i}}{\underset{k=1}{\sum }}\overset{q_{i}}{\underset{j=1}{\sum }}m_{ijk}ln\frac{m_{ijk}}{m_{ij}}-\frac{1}{2}\left(\ln N\right)\overset{n}{\underset{i=1}{\sum }}q_{i}(r_{i}-1)$$

### 2.3. Edge Types, Kinds and Types of the Experts’ Knowledge, and Experts’ Accuracies

#### 2.3.1. Edge Types

**Figure 1:**
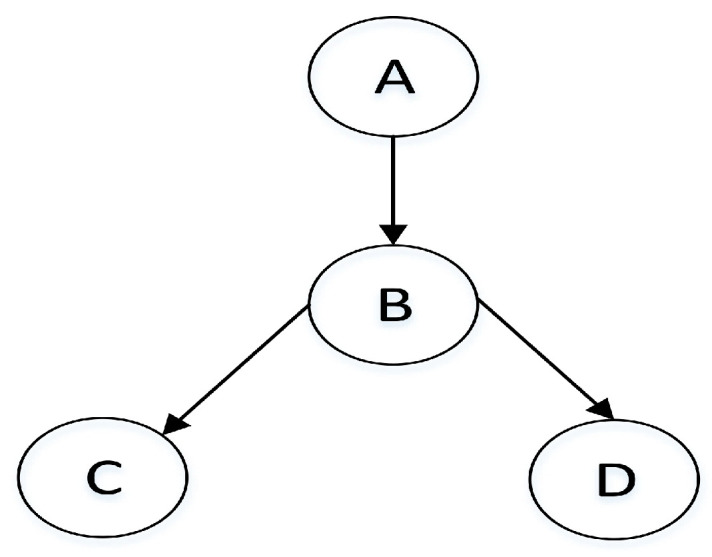
A simple Bayesian network structure.

Given the number of random variables in Bayesian network *n*, there exists $\frac{n\left(n-1\right)}{2}$ different node pairs. From Figure 1, since there are four nodes in this simple Bayesian network structure, the number of node pairs is $N=\frac{4\times (4-1)}{2}=6$.

If the *i*-th node pair is (X,Y), the state of the edge between node X and node Y is defined as $e_{i}$, where:

- $e_{i}=\to \mathrm{represents}<X,Y>\in E$,
- $e_{i}=\leftarrow \mathrm{represents}<Y,X>\in E$,
- $e_{i}=↮\mathrm{represents}<\emptyset >\in E$.

(X,Y) represents an unordered pair, and <X,Y> represents an ordered pair that node X is the starting point and node Y is the ending point. We can clearly see that there are three types of each edge in the structure: {→,←,↮}. As shown in Figure 1, the Bayesian network structure has 6 node pairs that are ordered as (A,B), (A,C), (A,D), (B,C), (B,D), (C,D), and there are $e_{1}=\to ,\;e_{2}=↮,\;e_{3}=↮,\;e_{4}=\to ,\;e_{5}=\to ,\;e_{6}=↮$, which can also represent {<A,B>, <B,C>, <B,D>}∈E.

#### 2.3.2. Kinds and Types of The Experts’ Knowledge, and Experts’ Accuracies

(1) The types of the experts’ knowledge and representation

In this paper, experts’ knowledge can be divided into two types: explicit knowledge and vague knowledge:

**Definition** **1.** Explicit knowledge is the knowledge that can make the causal relationship between nodes in BN structure clear.

**Definition** **2.** Vague knowledge is the knowledge that cannot make the causal relationship between nodes in BN structure clear.

We define the *i*-th expert’s knowledge of *j*-th node pair $V_{j}^{i}$ taking values in the domain {→,←,↮,−,↛,↚}, which builds upon the restrictions originally proposed by [24]. The main advantages of our kinds of experts’ knowledge are that we simplify kinds of experts’ knowledge to be easier to achieve and consider uncertainty of experts’ knowledge. Now let us formally define the six kinds of experts’ knowledge. Assuming that the *j*-th node pair is (X,Y):

**Definition** **3.** $V_{j}^{i}=\to$*is that the i-th expert deems that node*X*is the parent of node*Y*in the j-th node pair.*

**Definition** **4.** $V_{j}^{i}=\leftarrow$*is that the i-th expert deems that node*Y*is the parent of node*X*in the j-th node pair.*

**Definition** **5.** $V_{j}^{i}=↮$*is that the i-th expert deems that node*X*isn’t associated with node*Y*in the j-th node pair.*

**Definition** **6.** $V_{j}^{i}=-$*is that the i-th expert deems that node*X*is associated with node*Y*in the j-th node pair. However, it is uncertain whether the relationship between node X and node Y is*→*or*←.

**Definition** **7.** $V_{j}^{i}=↛$*is that the i-th expert deems that node X isn’t the parent of node Y in the j-th node pair. This means that it is uncertain whether the relationship between node X and node Y is ← or ↮.*

**Definition** **8.** $V_{j}^{i}=↚$*is that the i-th expert deems that*Y*node isn’t the parent of node*X*in the j-th node pair. This means that it is uncertain whether the relationship between node X and node Y is*→*or*↮.

According to the above definitions, there exists six kinds of experts’ knowledge in BN: {→,←,↮,−,↛,↚}. For convenience, we number the six kinds of experts’ knowledge with 1–6.

Obviously, explicit knowledge includes three kinds of experts’ knowledge: {→,←,↮}, and vague knowledge includes three kinds of experts’ knowledge: {↚,↛,−}.

In this paper, experts’ knowledge can be represented by sets. Explicit knowledge can be represented by a set *C* which consists of subsets of each expert’s explicit knowledge. The set of the *i*-th expert’s explicit knowledge $C^{i}$ consists of two subsets of node pairs: $C_{E}^{i}$ and $C_{A}^{i}$. $C_{E}^{i}$ is the set of knowledge which indicate the existence edge between the node pair, including {→,←}. $C_{A}^{i}$ is the set of knowledge which indicate the absent edge between the node pair, including {↮}. Each element $<x,y>\in C_{E}^{i}$ is associated with the corresponding $V_{j}^{i}=\to$, and each element $\left(x,y\right)\in C_{A}^{i}$ is associated with the corresponding $V_{j}^{i}=↮$. $C^{\prime }$ represents the set of explicit knowledge after fusion and the form is the same as $C^{i}$ including $C_{E}^{\prime }$ and $C_{A}^{\prime }$.

Vague knowledge can also be represented by an another set *I* which consists of subsets of each expert’s vague knowledge. The set of the *i*-th expert’s vague knowledge $I^{i}$ consists of two subsets of node pairs: $I_{E}^{i}$ and $I_{A}^{i}$. $V_{E}^{i}$ is the set of knowledge which indicate the existence edge between the node pair, including {−}. $I_{A}^{i}$ is the set of knowledge which indicate the absent edge between the node pair, including {↚,↛}. Each element $\left(x,y\right)\in I_{E}^{i}$ is associated with the corresponding $V_{j}^{i}=-$, and each element $<x,y>\in I_{A}^{i}$ is associated with the corresponding $V_{j}^{i}=↛$. $I^{\prime }$ represents the set of vague knowledge after fusion and the form is the same as $I^{i}\;including\;I_{E}^{\prime }$ and $I_{A}^{\prime }$.

(2) Experts’ accuracies

Considering the actual situation that the experts are heterogeneous. In this paper, the accuracy of an expert is represented by six parameters, which we extend the accuracy of an expert in the field of explicit knowledge [17] to the accuracy of an expert in the field of vague knowledge:

- $\gamma_{1}$: The probability of the correct orientation in the actual network in the field of explicit knowledge;
- $\gamma_{2}$: The probability of the reverse orientation in the actual network in the field of explicit knowledge;
- *γ*_3_: The probability of correctly detecting the absent edges in the actual network in the field of explicit knowledge;
- $\beta_{1}$: The probability of the correct orientation in the actual network in the field of vague knowledge;
- $\beta_{2}$: The probability of the reverse orientation in the actual network in the field of vague knowledge;
- $\beta_{3}$: The probability of correctly detecting the absent edges in the actual network in the field of vague knowledge.

From the above, there are two types of accuracy of an expert: the accuracy of an expert in the field of explicit knowledge and the accuracy of an expert in the field of vague knowledge. In this paper, we use a superscript such as $\gamma_{1}^{i},\gamma_{2}^{i},\gamma_{3}^{i},\beta_{1}^{i},\beta_{2}^{i},\beta_{3}^{i}$ to represent six accuracy parameters of the *i*-th expert.

## 3. Using Different Types of Experts’ Knowledge within the Hybrid Structure Learning Algorithm

In order to improve the learning performance and utilization of knowledge, this paper applies knowledge to two stage algorithm of hybrid algorithms respectively.

### 3.1. Using Different Types of Experts’ Knowledge with the First Stage Structure Learning Algorithm

In order to guide the search of the second stage structure learning algorithms better, we need to determine a better initial network structure in the first stage structure learning algorithms. In this paper, we formulate rules for adding and deleting edges of different types of experts’ knowledge to modify the initial network structure in order to improve the accuracy of the initial network structure and improve the overall performance of the algorithm. Firstly, the credibility of experts’ knowledge is determined according to the accuracy parameters of experts. In addition, the different experts’ knowledge is fused based on the credibility of experts’ knowledge. The purpose of these two steps is to fuse different experts’ knowledge on the same set of nodes based on credibility. Finally, by formulating rules for adding and deleting edges of different types of experts’ knowledge, the fusion of experts’ knowledge is modified the initial network structure based on credibility. It can be divided into the following three steps.

(1) To determine the credibility of experts’ knowledge

The credibility of experts’ knowledge is the probability that experts’ knowledge is true. Different kinds of experts’ knowledge have different credibility. The credibility θ of experts’ knowledge is determined according to the accuracy parameters of experts.

$\theta_{l}^{i}$ represents the credibility that the experts’ knowledge is the *l*-th kind and the expert is the *i*-th. Take the *i*-th expert as an example to explain the credibility of different kinds of expert knowledge $\theta_{l}^{i},l\in \left\{1,2,3,4,5,6\right\}$.

The *i*-th expert’ explicit knowledge which indicates the existence edge between the node pair includes {→,←}. The two kinds of knowledge don’t essentially differ and the credibility is the same, and credibility of two kinds of knowledge can be written as $\theta_{1}^{i}=\theta_{2}^{i}=\gamma_{1}^{i}$.

The *i*-th expert’ explicit knowledge which indicates the absent edge between the node pair includes {↮}, and credibility of this kind of knowledge can be written as $\theta_{3}^{i}=\gamma_{3}^{i}$.

The *i*-th expert’ vague knowledge which indicates the existence edge between the node pair includes {−}, and credibility of this kind of knowledge can be written as $\theta_{4}^{i}=\beta_{1}^{i}+\beta_{2}^{i}$.

The *i*-th expert’ vague knowledge which indicates the absent edge between the node pair includes {↛,↚}. The two kinds of knowledge don’t essentially differ and the credibility is the same, and credibility of two kinds of knowledge can be written as $\theta_{5}^{i}=\theta_{6}^{i}=1-\beta_{2}^{i}$.

(2) The different experts’ knowledge fusion based on the credibility of experts’ knowledge

As mentioned earlier, the *i*-th expert’s knowledge of *j*-th node pair $V_{j}^{i}$ takes values in the domain {→,←,↮,↚,↛,−}. For a specific node pair, the experts’ knowledge may not be inconsistent. We take the following four steps to fuse different experts’ knowledge for the *j*-th node pair:

(a) Divide the experts for the *j*-th node pair into six sets including $G_{1}^{j},G_{2}^{j},G_{3}^{j},G_{4}^{j},G_{5}^{j}$ and $G_{6}^{j}$ (Define $G_{l}^{j}$ as a set of experts for the *j*-th node pair with the same kind lth of knowledge).
(b) Sum and normalize the credibility of the six kinds of knowledge for the *j*-th node pair:
  (6) $$P_{l}^{j}=\frac{\sum_{i\in G_{l}^{j}}\theta_{l}^{i}}{\sum_{l\in \left\{1,2,3,4,5,6\right\}}\sum_{i\in G_{l}^{j}}\theta_{l}^{i}}$$
(c) Divide the close interval [0,1] into six subintervals including $\left[0,P_{1}^{j}\right)$, $\left[P_{1},P_{1}^{j}+P_{2}^{j}\right)$, $\left[P_{1}^{j}+P_{2}^{j},P_{1}^{j}+P_{2}^{j}+P_{3}^{j}\right)$, $\left[P_{1}^{j}+P_{2}^{j}+P_{3}^{j},P_{1}^{j}+P_{2}^{j}+P_{3}^{j}+P_{4}^{j}\right)$, $\left[P_{1}^{j}+P_{2}^{j}+P_{3}^{j}+P_{4}^{j},P_{1}^{j}+P_{2}^{j}+P_{3}^{j}+P_{4}^{j}+P_{5}^{j}\right)$ and $\left[P_{1}^{j}+P_{2}^{j}+P_{3}^{j}+P_{4}^{j}+P_{5}^{j},1\right]$. For a rand(1) which is a random number [0,1], if $rand\left(1\right)\in I_{k}$ where k is the *k*-th subinterval as previously described, choose the *l*-th kind of experts’ knowledge where l=k as the result of knowledge fusion for *j*-th node pair. These six kinds of knowledge are not mutually exclusive, so we cannot simply choose the knowledge with higher $P_{i}^{j}$. We divide the close interval [0,1] into six subintervals whose length are $P_{1}^{j}$, $P_{2}^{j}$, $P_{3}^{j}$, $P_{4}^{j}$, $P_{5}^{j}\;\mathrm{and}\;P_{6}^{j}$, which is the same with roulette. And we use a random number located in [0,1] which trends to choose the knowledge with higher $P_{i}^{j}$. Put the result into the set of *C*′ or $I^{\prime }$.
(d) Calculate the credibility $\theta_{k}^{j}$ of the result of knowledge fusion for the *j*-th node pair:
  (7) $$\theta_{k}^{j}=\frac{\sum_{i\in G_{k}^{j}}\theta_{k}^{i}}{card\left(G_{k}^{j}\right)}$$
  where $card\left(G_{k}^{j}\right)$ is a function that returns the number of elements in the set $G_{k}^{j}$. Put the $\theta_{k}^{j}$ into the set of credibility *B.* The experts’ knowledge set $C^{\prime }$ and $I^{\prime }$ and the corresponding credibility set *B* after fusion will be used as inputs to the following rule.

(3) Modify the process of generating the initial network structure by rules for adding and deleting edges of different types of experts’ knowledge

In this paper, we use the rule-based method to formulate the rules for adding and deleting edges of different types of experts’ knowledge. Based on the rules, we modify the undirected network structure that is determined by the first process in the first stage structure learning and the partial directed network structure that is determined by the second process in the first stage structure learning. The rules for adding and deleting edges of different types of experts’ knowledge are described below.

Rules for adding and deleting edges of vague knowledge include the rule for adding the edge in the undirected network structure and the rule for deleting the edge in the partial directed network structure.

**Rule** **1.** *(The rule for adding the edge in the undirected network structure)*

$H^{\prime }=\left(V,E_{H}^{\prime }\right)$ *represents the undirected network structure after adding the edge, and* $H=\left(V,E_{H}\right)$ *represents the undirected network structure before adding the edge. For* $\left(X,Y\right)\in I_{E}^{\prime }$ *, including* {−} *, the credibility of knowledge is* $\theta_{4}^{j}$ *, if* $rand\left(1\right)\in \left(0,\theta_{4}^{j}\right)$ *,* $E_{H}^{\prime }=E_{H}\cup^{\text{​}}\left(X,Y\right)$ *.*

**Rule** **2.** (The rule for deleting the edge in the partial network structure)

$H^{\prime }=\left(V,E_{H}^{\prime }\right)$ *represents the partial network structure after deleting the edge, and* $H=\left(V,E_{H}\right)$ *represents the partial network structure before deleting the edge. For* $X,Y\in I_{A}^{\prime }$ *, including* {↚,↛} *, the credibility of knowledge is* $\theta_{l}^{j}$ *, with l = 5 or 6, if* $rand\left(1\right)\in \left(0,\theta_{l}^{j}\right)$ *,* $E_{H}^{\prime }=E_{H}\backslash \langle X,Y\rangle$ *.*

*Rules for adding and deleting edges of explicit knowledge include the rule for deleting the edge in the undirected network structure and the rule for adding the edge in the partial directed network structure.*

**Rule** **3.** (The rule for deleting the edge in the undirected network structure)

$H^{\prime }=\left(V,E_{H}^{\prime }\right)$*represents the undirected network structure after deleting the edge, and*$H=\left(V,E_{H}\right)$*represents the undirected network structure before deleting the edge. For*$\left(X,Y\right)\in C_{A}^{\prime }$*, including*{↮}, *the credibility of knowledge is*$\theta_{3}^{j}$*, if*$rand\left(1\right)\in \left(0,\theta_{3}^{j}\right)$*,*$E_{H}^{\prime }=E_{H}\backslash \left(X,Y\right)$*.*

**Rule** **4.** (The rule for adding the edge in the partial network structure)

$H^{\prime }=\left(V,E_{H}^{\prime }\right)$*represents the partial network structure after adding the edge, and*$H=\left(V,E_{H}\right)$*represents the partial network structure before adding the edge. For*$X,Y\in C_{E}^{\prime }$*, including*{→,←}*, the credibility of knowledge is*$\theta_{l}^{j}$*, with l = 1 or 2, if*$\mathrm{rand}(1)\in (0,\theta_{l}^{j}),\;{E^{\prime }}_{H}=E_{H}\cup \langle X,Y\rangle$.

**Figure 2:**
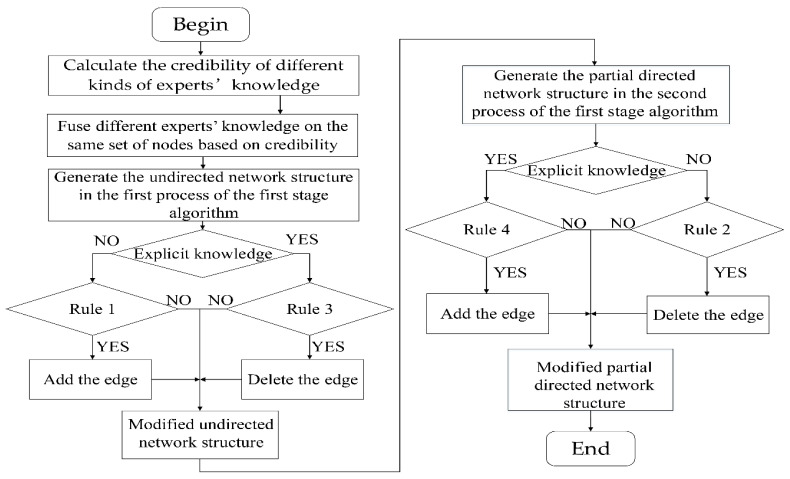
The schematic diagram of using different types of experts’ knowledge with the first stage structure learning algorithm.

The schematic diagram of using different types of experts’ knowledge with the first stage structure learning algorithm is shown in Figure 2.

### 3.2. Using Different Types of Experts’ Knowledge with the Second Stage Structure Learning Algorithm

The second stage structure learning algorithms use a search strategy to select the structure with the highest score of a scoring function. The key to select the “optimal” structure is the scoring function.

Explicit-accuracy-based scoring function is proposed by [17]. First, [17] sets up three various problem models. Then, [17] derives some independence statements from three models using the principle of d-separation. Last, scoring function is derived with simplification of independence statements derived above. And this scoring function is given as:

(8) $$Score_{explicit}=logP\left(D|G\right)+\overset{R}{\underset{j=1}{\sum }}\overset{N}{\underset{i=1}{\sum }}logP(V_{i}^{j}|e_{i},\gamma^{j})$$

**Figure 3:**
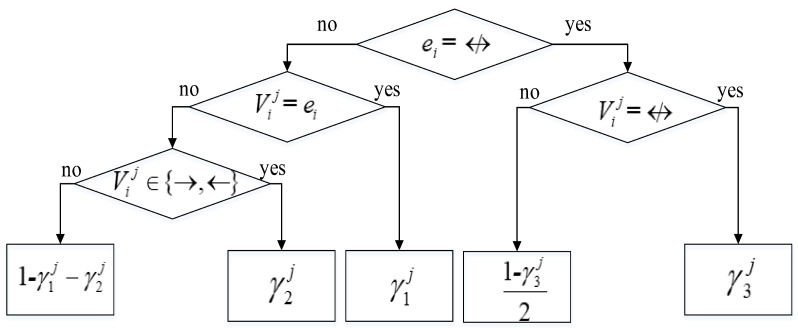
Decision tree for computing $P(V_{i}^{j}|e_{i},\gamma^{j})$.

This scoring function is composed of two parts: logP(D|G) and $\sum_{j=1}^{R}\sum_{i=1}^{N}logP(V_{i}^{j}|e_{i},\gamma^{j})$. The first part is the marginal likelihood part of BDeu scoring function [18]. $P(V_{i}^{j}|e_{i},\gamma^{j})$ in the second part indicates the probability of $V_{i}^{j}$ given $e_{i}\mathrm{and}\gamma^{j}$ and is computed using the decision tree shown in Figure 3.

We can comprehend this scoring function in this way. The first part of Explicit-accuracy-based scoring function means the scoring function which measures the goodness of network to data. The second part of Explicit-accuracy-based scoring function means a penalization which measures the difference between experts’ knowledge with network. Experts’ knowledge here only refers to the explicit knowledge. Based on this, in this paper, the Explicit-Vague-BIC (EVBIC) scoring function is proposed and given as:

(9) $$Score_{EVBIC}=BIC\left(D\right)+\overset{R}{\underset{j=1}{\sum }}\overset{N\_explicit}{\underset{i=1}{\sum }}logP(V_{explicit}{}_{i}^{j}|e_{i},\gamma^{j})+k\overset{R}{\underset{j=1}{\sum }}\overset{N\_vague}{\underset{i=1}{\sum }}logP(V_{vague}{}_{i}^{j}|e_{i},\beta^{j}),$$

where N_explicit is the number of node pairs with explicit knowledge, and N_vague is the number of node pairs with vague knowledge. $V_{explicit}{}_{i}^{j}$ is the three kinds of explicit knowledge and $V_{vague}{}_{i}^{j}$ is the three kinds of vague knowledge. The EVBIC scoring function is composed of three parts. In the first part, we use the BIC scoring function as the scoring function which measures the goodness of network to data. The second and third part represent penalizations which measure the difference between experts’ knowledge with network. The second part represents the penalization which measures the difference between explicit knowledge and network. The third part represent the penalization which measures the difference between vague knowledge and network. In the same case, the vague knowledge is more ambiguous and weaker for building network structure than explicit knowledge. Therefore, we consider using a coefficient k∈(0,1) to weigh the contribution of explicit knowledge and vague knowledge. Considering each vague knowledge corresponds to two kinds of relationship between nodes in BN structure, in this paper, we take k=0.5.

**Figure 4:**
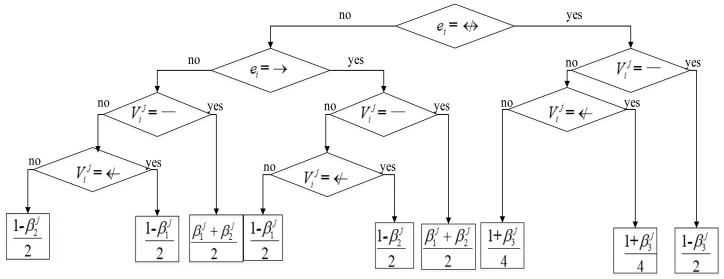
Decision tree for calculating $P(V_{vague}{}_{i}^{j}|e_{i},\beta^{j})$.

The term $P(V_{explicit}{}_{i}^{j}|e_{i},\gamma^{j})$ in the second part in Equation (9) is calculated as the decision tree shown in Figure 3. The calculation of the term $P(V_{vague}{}_{i}^{j}|e_{i},\beta^{j})$ in the third part in Equation (9) is same as Figure 3, as shown in Figure 4.

## 4. Experiments

### 4.1. Experimental Setup

All the simulation work is implemented and executed in MATLAB 2016b, using the Bayesian network toolbox FullBNT-1.0.7, the maximum information coefficient toolbox minepy-1.2.1 and the graph toolbox matgraph-2.0. The PC has a CPU of Intel 3.40 GHz, an 8 GB memory and Windows 10 operating system.

**Figure 5:**
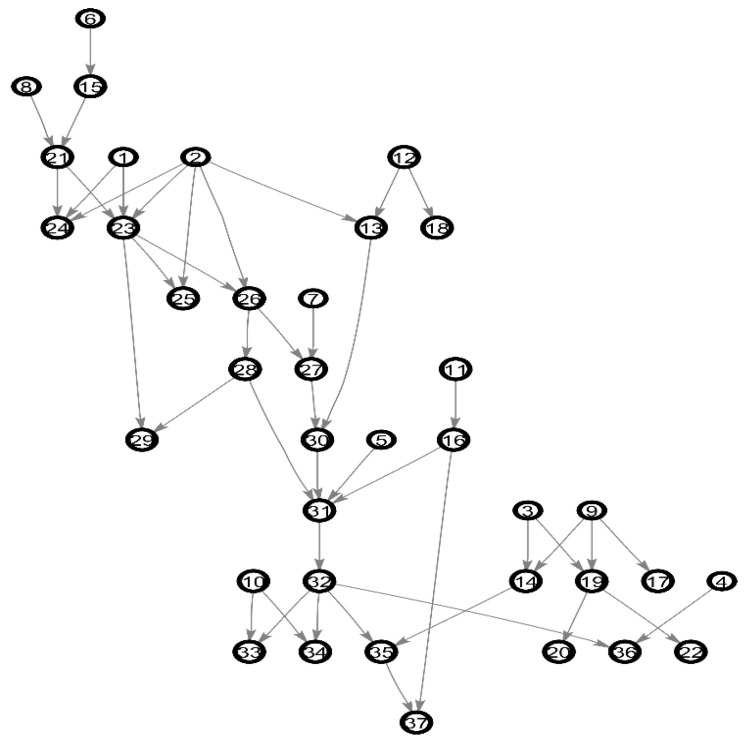
The Alarm network.

**Figure 6:**
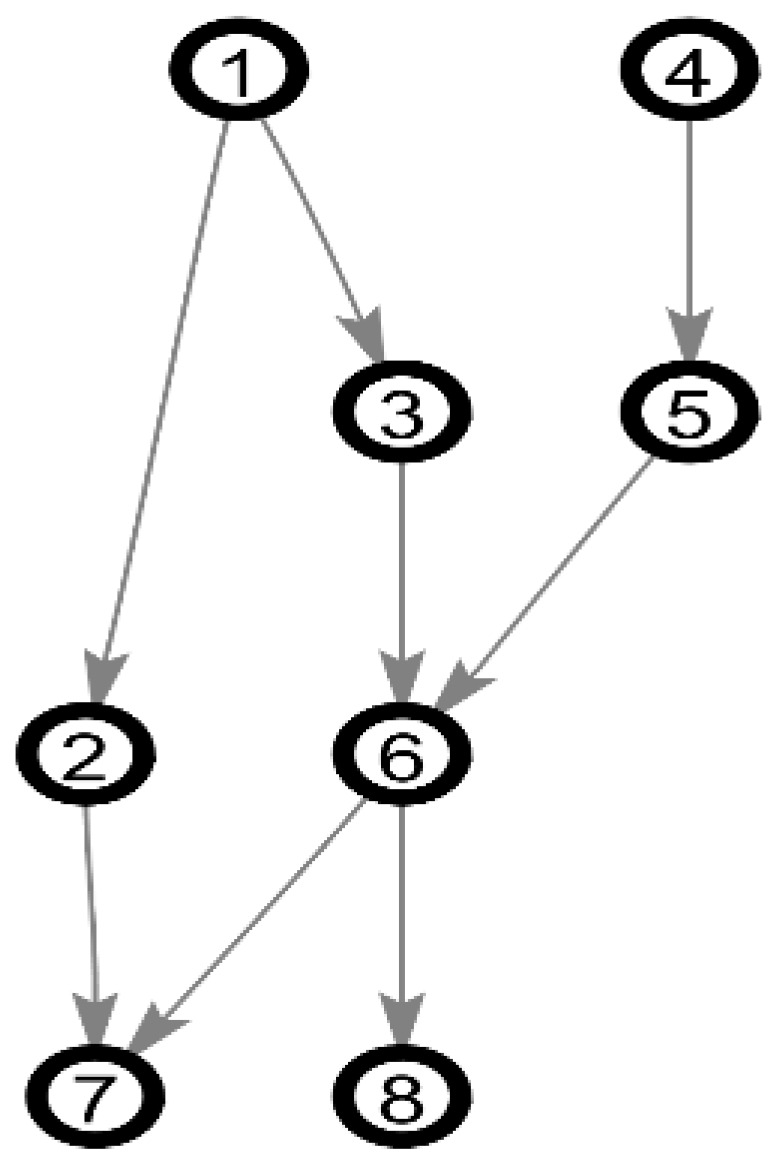
The Asia network.

We use the well-known benchmarks of BN: Alarm and Asia as the experimental network. The Alarm network is a medical diagnostic system for patient monitoring, with 37 nodes and 46 arcs. Because of the complexity of the network structure, it is considered as a standard network to measure the quality of BN structure learning. The Asia network is a small Bayesian network for virtual medical cases, with 8 nodes and 8 arcs. Figure 5 and Figure 6 shows the structure of the Alarm network and Asia network. The experiment network mainly uses Alarm network, the default network.

We measure the operators to reconstruct the original network from the learned network, including the number of deleted arcs (D), the number of added arcs (A) and the number of inverted arcs (I). The total number of the three operators is called Structural Hamming Distance (SHD). Measuring the same edges between the learned network and the original network is called Correct Edges (C). The smaller A, D, I and SHD are, the better the structure learning is. The bigger C is, the better the structure learning is. The measures of performance are: (1) BIC scoring function, which also guides the score-based structure learning algorithms. The higher the BIC score is, the better the structure learning is. (2) The measures of the structural difference between the learned and original networks such as A, D, I, SHD and C. In the experiments, we consider E=10 and corresponding ten groups of experts’ accuracies. Table 1 lists the details of ten group parameters of experts’ accuracies.

In this paper, we use a parameter v∈[0,1] as the percentage of experts’ knowledge. In this paper, the value of the parameter v is selected from {0.4,0.5,0.6}. We use the hybrid structure learning algorithm of MIC-BPSO with BIC scoring function to learn the network structure by the data and three different cases of experts’ knowledge. Our experiments are divided into three cases for the Results and Discussion. For the first case, we use three different cases of experts’ knowledge in the first stage of the hybrid algorithm in order to verify the validity of using the different types of experts’ knowledge in the first stage algorithm. For the second case, we use three different cases of experts’ knowledge in the second stage of the hybrid algorithm in order to verify the validity of using the different types of experts’ knowledge in the second stage algorithm. For the third case, we use three different cases of experts’ knowledge in two stage of the hybrid algorithm in order to verify the validity of using the different types of experts’ knowledge in the hybrid algorithm.

In the experiments, we have three cases of experts’ knowledge:

(1) Explicit knowledge: this case of experts’ knowledge only uses explicit knowledge in BN.
(2) Vague knowledge: this case of experts’ knowledge only uses vague knowledge in BN.
(3) EV knowledge: this case of experts’ knowledge uses the mixed knowledge including explicit knowledge and vague knowledge in BN.

In our experiments, we use 10 different datasets with 2000 samples and 10 different datasets with 5000 samples in the Alarm network and we use 10 different datasets with 500 samples and 10 different datasets with 2000 samples in the Asia network. For each tuple {case of experts’ knowledge, v}, we generate 10 different experts’ knowledge sets. In each experiment, we use one dataset and one experts’ knowledge set to learn the BN structure.

### 4.2. Results and Discussion

#### 4.2.1. Results and Discussion Using the Different Types of Experts’ Knowledge in the First Stage Algorithm

We calculate the A, D, I and SHD of the learned network. MR is the initial Mean Result, representing 10 runs of the average results. BR is the initial Best Result, representing 10 runs of the optimal value. The term data means using the hybrid algorithm to learn structure without experts’ knowledge and is equivalent to v=0. Table 2 shows the results of this experiment.

The smaller A, D, I and SHD are, the better the structure learning is. The SHD shows the total difference between the learned and original networks and A, D, I represent the details of different kinds of difference between the learned and original networks. From Table 2, we can easily find that the results of learning with each case of experts’ knowledge are better than the results of learning without experts’ knowledge, which proves that the proposed rules using different types of experts’ knowledge in the first algorithm is effective. Compared with three cases of experts’ knowledge, the experimental results indicate that learning with explicit knowledge and vague knowledge is better than learning with explicit knowledge alone.

Because the proposed method in the first stage structure learning algorithm takes no account of BIC scoring function, we can compare the learned network with experts’ knowledge with the learned network with data by calculating BIC scoring function. Table 3 and Table 4 show the results of this experiment.

The higher BIC score is, the better the structure learned is. From Table 3 and Table 4, we can see that the BIC scores of learning with each case of experts’ knowledge are higher than the BIC scores of learning without experts’ knowledge. It is shown that the proposed rules using different types of experts’ knowledge in the first algorithm is effective. Comparing with three cases of experts’ knowledge, we can easily find that learning with explicit knowledge and vague knowledge is better than learning with explicit knowledge alone.

#### 4.2.2. Results and Discussion Using the Different Types of Experts’ Knowledge in the Second Stage Algorithm

We calculate the A, D, I and SHD of learned networks. Table 5 shows the results of this experiment.

From Table 5, we can easily find that the results of learning with each case of experts’ knowledge are better than the results of learning without experts’ knowledge. It is shown that the proposed the EVBIC scoring function using different types of experts’ knowledge in the second algorithm is effective. Comparing with three cases of experts’ knowledge, we can easily find that learning with explicit knowledge and vague knowledge is better than learning with explicit knowledge alone.

**Figure 7:**
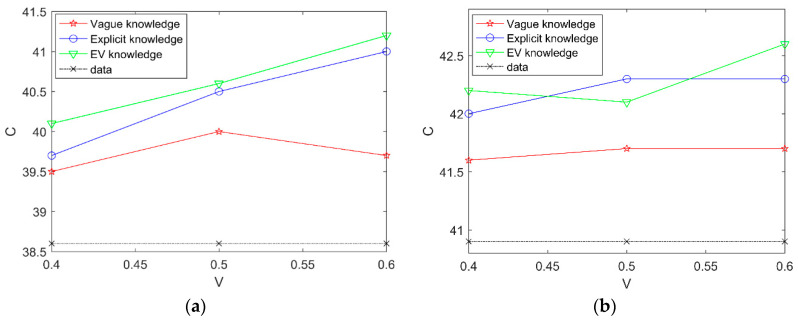
The correct edges of learned networks with different datasets in the second Results and Discussion case; (**a**) the correct edges of learned networks with 2000 samples in the second Results and Discussion case; (**b**) the correct edges of learned networks with 5000 samples in the second Results and Discussion case.

We calculate the correct edges of learned networks. Figure 7 shows the results of this experiment.

In Figure 7, the horizontal coordinate represents the percentage of experts’ knowledge and the vertical coordinate represents average SHD. Compared to learning with three cases of experts’ knowledge and learning with data alone in different datasets, the experimental results indicate that the proposed the EVBIC scoring function using different types of experts’ knowledge in the second algorithm is effective and learning with explicit knowledge and vague knowledge is better than learning with explicit knowledge alone.

#### 4.2.3. Results and Discussion Using the Different Types of Experts’ Knowledge in the Hybrid Algorithm

In order to ensure the robustness of the results, we calculate the A, D, I and SHD of learned networks for the Asia network and the Alarm network. Table 6 and Table 7 show the results of this experiment.

The smaller A, D, I and SHD are, the better the structure learning is. The SHD shows the total difference between the learned and original networks and A, D, I represent the details of different kinds of difference between the learned and original networks. From Table 6, we can easily find that the results of learning with each case of experts’ knowledge in the Alarm network are better than the results of learning without experts’ knowledge in the Alarm network. It is shown that the hybrid algorithm using different types of experts’ knowledge are effective. Comparing with three cases ofexperts’ knowledge, we can easily see that learning with explicit knowledge and vague knowledge is better than learning with explicit knowledge alone. From Table 7, according to the Asia network, we can also find that the hybrid algorithm using different types of experts’ knowledge are effective. It is thus proved that the result is robust.

From Table 7, according to the Asia network, we can also find that the hybrid algorithm using different types of experts’ knowledge are effective. It is thus proved that the result is robust.

**Figure 8:**
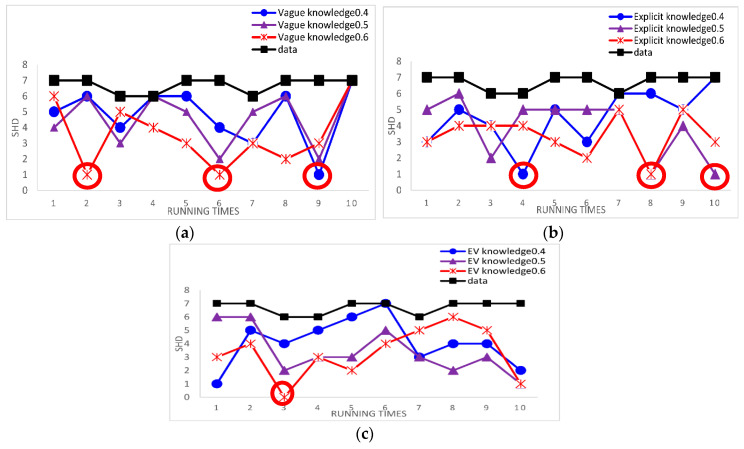
The single SHD of learned networks using different case of experts’ knowledge and using data alone for the Alarm network in the third Results and Discussion case. (**a**) The single SHD of learned networks using vague knowledge and using data alone in 5000 samples; (**b**) the single SHD of learned networks using explicit knowledge and using data alone in 5000 samples; (**c**) the single SHD of learned networks using EV knowledge and using data alone in 5000 samples.

**Figure 9:**
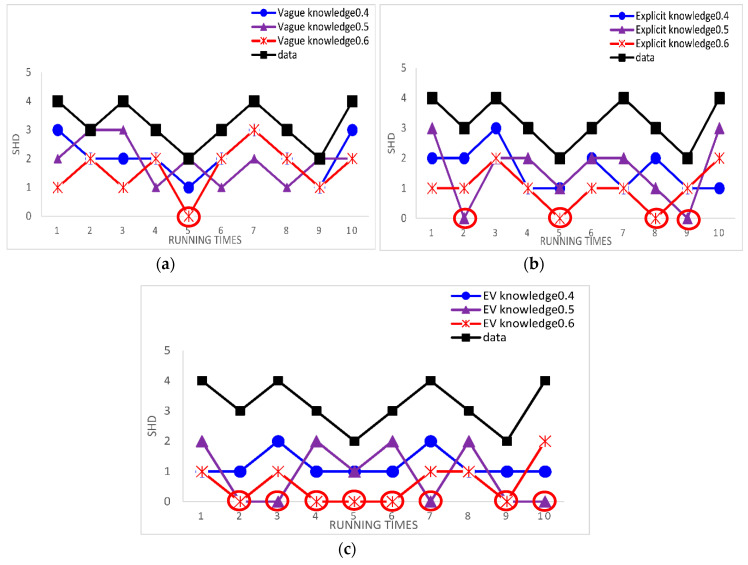
The single SHD of learned networks using different case of experts’ knowledge and using data alone for the Asia network in the third Results and Discussion case. (**a**) The single SHD of learned networks using Vague knowledge and using data alone in 500 samples; (**b**) the single SHD of learned networks using Explicit knowledge and using data alone in 500 samples; (**c**) the single SHD of learned networks using EV knowledge and using data alone in 500 samples.

In order to ensure the robustness of the results, we use the Asia network and Alarm network as our standard networks. Compared to learning with three cases of experts’ knowledge and learning with data alone by calculating the single SHD, the experimental results can directly the effect of structure learning. Because the results of using the samples = 5000 is the same as the samples = 2000 for the Alarm network and the results of using the samples = 500 is the same as the samples = 2000 for the Asia network, we use samples = 5000 for the Alarm network and samples = 500 for the Asia network as examples. Figure 8 and Figure 9 show the results of this experiments.

The red circles mark the best result. The smaller SHD is, the better the structure learning is. From Figure 8 and Figure 9, we can easily see that the best results appear in learning BN structure with knowledge and the learned BN structure is more similar to the original network structure through using knowledge than through using data alone. From Figure 8, according to the Alarm network, the results indicate that our proposed method using the experts’ knowledge in the hybrid algorithm is effective. From Figure 9, according to the Asia network, we can also get same result. It is proved that the results are robust.

**Figure 10:**
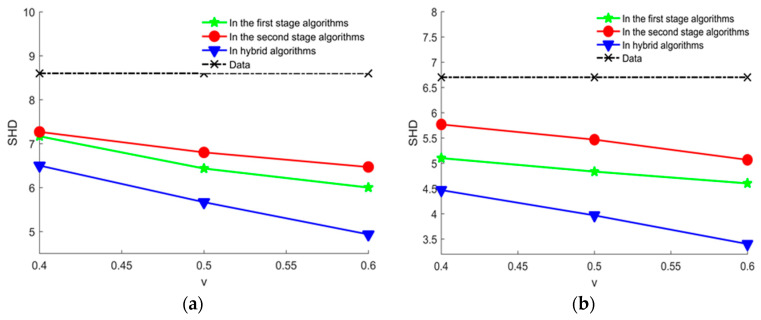
The average SHD values of learned networks using experts’ knowledge in different stage of the hybrid algorithm and learning with data alone in different datasets. (**a**) Average SHD values of learned networks using experts’ knowledge in different stage of the hybrid algorithm and learning with data alone in 2000 samples; (**b**) the average SHD values of learned networks using experts’ knowledge in different stage of the hybrid algorithm and learning with data alone in 5000 samples.

Compared learning with experts’ knowledge in different stage of the hybrid algorithm and learning with data alone by calculating average SHD, the experimental results are shown in Figure 10, where we can see that learning with experts’ knowledge can get a better structure than learning with data alone, which proved that our proposed method using the experts’ knowledge in the hybrid algorithm is effective. Moreover, learning with experts’ knowledge in two stage of the hybrid algorithm can get a better structure than learning with experts’ knowledge in each stage of the hybrid algorithm. It is indicated that using experts’ knowledge in each stage of the hybrid algorithm is not redundant. It is shown that our proposed method using experts’ knowledge is effective.

**Figure 11:**
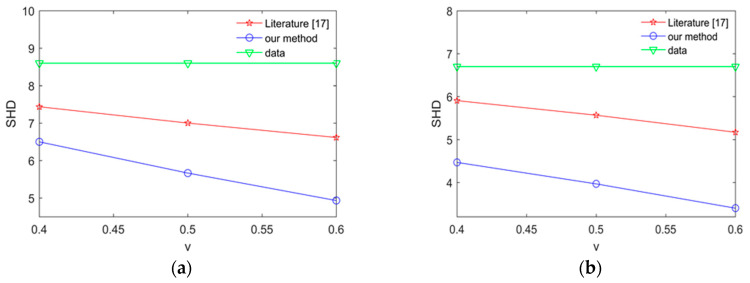
The average SHD values for the Alarm network using different methods. (**a**) The average SHD values for the Alarm network using different method in 2000 samples; (**b**) the average SHD values for the Alarm network using different method in 5000 samples.

**Figure 12:**
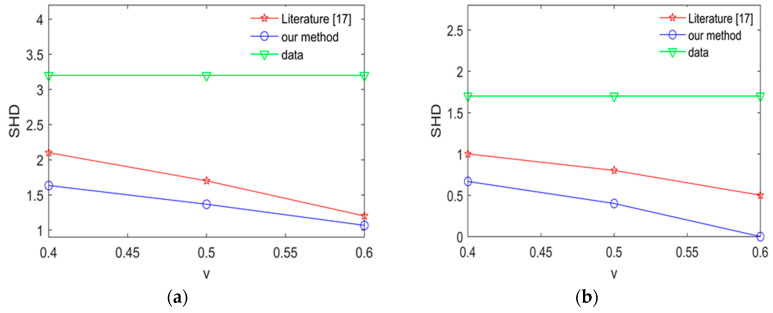
The average SHD values for the Asia network using different method. (**a**) The average SHD values for the Asia network using different method in 2000 samples; (**b**) the average SHD values for Asia network using different method in 5000 samples.

To expose the effectiveness of proposed method, we compare our method with the method in [17] by calculating average SHD in the Alarm network and Asia network. the experimental results are shown Figure 11 and Figure 12.

The smaller SHD values are, the better the structure learning is. From Figure 11 and Figure 12, we can easily find that the learned network using our proposed method is better than the learned network using the method in [17] in the Asia network and Alarm network. The results show that our proposed method in this paper has better learning performance and better learning effect.

## 5. Conclusions

We introduce a new method of using explicit knowledge and vague knowledge based on a hybrid structure learning algorithm for Bayesian network. The simulation experiment results show that our method achieves a higher BIC score and a smaller structure difference than previous algorithms by taking two types of experts’ knowledge into account. It is acknowledged that the introduction of explicit knowledge can improve the accuracy of Bayesian network structure based on data learning algorithm. Furthermore, we point out that the experts can give not only explicit knowledge but also vague knowledge in the real world and it is effective to improve the accuracy of Bayesian network structure by using vague knowledge. The main novelty of our proposed algorithm is that we consider these two types of experts’ knowledge in the process of learning Bayesian network structure but not explicit knowledge alone. Our proposed algorithm can also solve the problems of expert level difference and opinion conflict on the same group of node pairs.

## Figures and Tables

**Table 1:** The accuracy parameters assigned to experts.

	$\gamma_{i}^{j},\beta_{i}^{j}$	$\gamma_{1}^{j},\beta_{1}^{j}$	$\gamma_{2}^{j},\beta_{2}^{j}$	$\gamma_{3}^{j},\beta_{3}^{j}$
*E*				
1		0.65	0.15	0.7
2		0.65	0.16	0.75
3		0.67	0.15	0.78
4		0.7	0.17	0.77
5		0.75	0.14	0.8
6		0.78	0.11	0.81
7		0.8	0.12	0.82
8		0.72	0.2	0.79
9		0.79	0.18	0.8
10		0.8	0.13	0.8

**Table 2:** A, D, I and SHD values of learned networks in the first Results and Discussion case.

The Case of Knowledge	Measures of Performance		2000				5000			
			0.4	0.5	0.6	Data	0.4	0.5	0.6	Data
Vague knowledge	MR	SHD	7.5	6.9	7	8.6	5.4	5.3	5.2	6.7
		D	2.9	2.6	2.4	3.1	1.9	1.7	2.1	2.1
		A	1	0.9	0.7	1.2	1	1	1.1	1.6
		R	3.6	3.4	3.9	4.3	2.5	2.6	2.2	3
	BR	SHD	5	5	5	7	2	2	3	6
Explicit knowledge	MR	SHD	7.3	6.4	5.6	8.6	5.1	4.6	4.5	6.7
		D	2.9	2.6	2.6	3.1	1.9	1.6	1.8	2.1
		A	1	0.9	0.6	1.2	1.1	0.9	0.8	1.6
		R	3.4	2.9	2.4	4.3	2.1	2.1	1.9	3
	BR	SHD	5	4	4	7	2	2	2	6
EV knowledge	MR	SHD	6.7	6	5.4	8.6	4.8	4.6	4.1	6.7
		D	2.8	2.7	2.6	3.1	1.7	1.4	1.2	2.1
		A	0.8	0.6	0.6	1.2	1	0.7	0.7	1.6
		R	3.1	2.7	2.2	4.3	2.1	2.5	2.2	3
	BR	SHD	5	3	3	7	3	2	2	6

**Table 3:** BIC score of learned networks with 2000 samples in the first Results and Discussion case.

The Case of Knowledge	Measures of Performance		2000			
			0.4	0.5	0.6	Data
Vague knowledge	MR	BIC score	−20,206.9	−20,202.5	−20,108.5	−20,288.4
	BR	BIC score	−19,900.5	−19,870.6	−19,472	−19,929
Explicit knowledge	MR	BIC score	−20,013.2	−19,904.8	−19829.2	−20,288.4
	BR	BIC score	−18,925.7	−18,903.8	−18,212.9	−19,929
EV knowledge	MR	BIC score	−19,813.5	−19,776.23	−19,738.14	−20,288.4
	BR	BIC score	−18,212.94	−18,212.94	−17,893.82	−19,929

**Table 4:** BIC score of learned networks with 5000 samples in the first Results and Discussion case.

The Case of Knowledge	Measures of Performance		5000			
			0.4	0.5	0.6	Data
Vague knowledge	MR	BIC score	−48,571.2	−48,544	−48,467.7	−48,689.4
	BR	BIC score	−48,044.4	−47,932.4	−47,581.1	−48,200.4
Explicit knowledge	MR	BIC score	−48,390	−48,095.6	−48,083.5	−48,689.4
	BR	BIC score	−47,886.6	−46,305	−47,000.3	−48,200.4
EV knowledge	MR	BIC score	−47,923.74	−47,823.73	−47,813.43	−48,689.4
	BR	BIC score	−46,682.75	−46,305	−45,768.92	−48,200.4

**Table 5:** A, D, I and SHD values of learned networks in the second Results and Discussion case.

The Case of Knowledge	Measures of Performance		2000				5000			
			0.4	0.5	0.6	Data	0.4	0.5	0.6	Data
Vague knowledge	MR	SHD	7.7	7.6	7.1	8.6	6.1	5.8	5.5	6.7
		D	2.8	2.9	3	3.1	2.2	2.1	2	2.1
		A	1	1	1	1.2	1.5	1.5	1.4	1.6
		R	3.9	3.7	3.1	4.3	2.4	2.2	2.1	3
	BR	SHD	7	7	6	7	5	5	5	6
Explicit knowledge	MR	SHD	7.4	6.8	6.4	8.6	5.8	5.4	5	6.7
		D	3.1	2.8	2.7	3.1	2.2	2.2	2.1	2.1
		A	0.9	0.9	0.9	1.2	1.3	1.2	1.2	1.6
		R	3.4	3.1	2.8	4.3	2.3	2	1.7	3
	BR	SHD	5	4	3	7	3	4	3	6
EV knowledge	MR	SHD	6.7	6	5.9	8.6	5.4	5.2	4.7	6.7
		D	2.7	2.9	2.8	3.1	2.1	2.4	2.1	2.1
		A	0.8	0.9	0.9	1.2	1.2	1	1	1.6
		R	3.2	2.2	2.2	4.3	2.1	1.8	1.6	3
	BR	SHD	3	4	3	7	4	3	2	6

**Table 6:** A, D, I and SHD values of learned network for the Alarm network in the third Results and Discussion case.

The Case of Knowledge	Measures of Performance		2000				5000			
			0.4	0.5	0.6	Data	0.4	0.5	0.6	Data
Vague knowledge	MR	SHD	7.3	6.8	6.1	8.6	4.8	4.6	3.5	6.7
		D	3	2.7	2.1	3.1	1.7	1.7	1.4	2.1
		A	1	0.8	0.5	1.2	1	0.9	0.8	1.6
		R	3.3	3.3	3.5	4.3	2.1	2	1.3	3
	BR	SHD	6	3	3	7	1	2	1	6
Explicit knowledge	MR	SHD	6.2	5.2	4.7	8.6	4.5	3.9	3.4	6.7
		D	2.6	2.4	2.4	3.1	2	1.7	1.5	2.1
		A	0.8	0.5	0.4	1.2	0.8	0.9	0.9	1.6
		R	2.8	2.3	1.9	4.3	1.7	1.3	1	3
	BR	SHD	3	3	3	7	1	1	1	6
EV knowledge	MR	SHD	6	5	4	8.6	4.1	3.4	3.3	6.7
		D	2.6	2.4	2.1	3.1	1.9	1.7	1.5	2.1
		A	0.7	0.4	0.1	1.2	1	0.8	0.9	1.6
		R	2.7	2.2	1.8	4.3	1.2	0.9	0.9	3
	BR	SHD	3	3	3	7	1	1	0	6

**Table 7:** A, D, I and SHD values of learned network for the Asia network in the third Results and Discussion case.

The Case of Knowledge	Measures of Performance		2000				5000			
			0.4	0.5	0.6	Data	0.4	0.5	0.6	Data
Vague knowledge	MR	SHD	2.1	1.9	1.6	3.2	0.9	0.6	0.4	1.7
		D	1.2	1.1	0.9	1.6	0.6	0.5	0.2	0.5
		A	0.8	0.5	0.3	1.2	0.3	0.1	0.1	0.7
		R	0.1	0.3	0.4	0.4	0.1	0	0.1	0.5
	BR	SHD	1	1	0	2	0	0	0	0
Explicit knowledge	MR	SHD	1.6	1.3	1	3.2	0.7	0.4	0.2	1.7
		D	0.9	1.0	0.6	1.6	0.4	0.2	0.1	0.5
		A	0.5	0.2	0.2	1.2	0.1	0.1	0	0.7
		R	0.2	0.1	0.2	0.4	0.2	0.1	0.1	0.5
	BR	SHD	1	0	0	2	0	0	0	0
EV knowledge	MR	SHD	1.2	0.9	0.6	3.2	0.4	0.2	0	1.7
		D	0.8	0.5	0.4	1.6	0.3	0.1	0	0.5
		A	0.3	0.2	0.1	1.2	0.1	0	0	0.7
		R	0.1	0.2	0.1	0.4	0	0.1	0	0.5
	BR	SHD	1	0	0	2	0	0	0	0
